# What influences national and foreign physicians’ geographic distribution? An analysis of medical doctors’ residence location in Portugal

**DOI:** 10.1186/1478-4491-10-12

**Published:** 2012-07-02

**Authors:** Giuliano Russo, Paulo Ferrinho, Bruno de Sousa, Cláudia Conceição

**Affiliations:** 1 International Public Health and Biostatistics Unit, Instituto de Higiene e Medicina Tropical, Universidade Nova de Lisboa and Centre for Malaria and Tropical Diseases, Associated Laboratory, Lisbon, Portugal; 2 Life and Health Sciences Research Institute (ICVS), School of Health Sciences, University of Minho, Braga, Portugal; 3 ICVS/3B’s - PT Government Associate Laboratory, Braga/Guimarães, Portugal

## Abstract

**Background:**

The debate over physicians’ geographical distribution has attracted the attention of the economic and public health literature over the last forty years. Nonetheless, it is still to date unclear what influences physicians’ location, and whether foreign physicians contribute to fill the geographical gaps left by national doctors in any given country. The present research sets out to investigate the current distribution of national and international physicians in Portugal, with the objective to understand its determinants and provide an evidence base for policy-makers to identify policies to influence it.

**Methods:**

A cross-sectional study of physicians currently registered in Portugal was conducted to describe the population and explore the association of physician residence patterns with relevant personal and municipality characteristics. Data from the Portuguese Medical Council on physicians’ residence and characteristics were analysed, as well as data from the National Institute of Statistics on municipalities’ population, living standards and health care network. Descriptive statistics, chi-square tests, negative binomial and logistic regression modelling were applied to determine: (a) municipality characteristics predicting Portuguese and International physicians’ geographical distribution, and; (b) doctors’ characteristics that could increase the odds of residing outside the country’s metropolitan areas.

**Results:**

There were 39,473 physicians in Portugal in 2008, 51.1% of whom male, and 40.2% between 41 and 55 years of age. They were predominantly Portuguese (90.5%), with Spanish, Brazilian and African nationalities also represented. *Population*, *Population’s Purchasing Power*, *Nurses per capita* and *Municipality Development Index* (MDI) were the municipality characteristics displaying the strongest association with national physicians’ location. For foreign physicians, the MDI was not statistically significant, while municipalities’ foreign population applying for residence appeared to be an additional positive factor in their location decisions. In general, being foreigner and male resulted to be the physician characteristics increasing the odds of residing outside the metropolitan areas. However, among the internationals, older doctors were more likely to reside outside metropolitan areas. Being Spanish or Brazilian (but not of African origin) was found to increase the odds of being based outside the Lisbon and Oporto metropolitan areas.

**Conclusions:**

The present study showed the relevance of studying one country’s physician population to understand the factors driving national and international doctors’ location decisions. A more nuanced understanding of national and foreign doctors’ location appears to be needed to design more effective policies to reduce the imbalance of medical services across geographical areas.

## Introduction

The debate over physicians’ practice location and geographical distribution has attracted the attention of both the economic and public health literature over the last forty years [1]. The issue of uneven distribution of health professionals worldwide has long been recognised [2], and much discussion exists on the most effective strategies to attract and retain physicians in rural and underserved areas [3]. In Portugal universal health coverage is offered through the private and public service free of charge at the point of use [4]. The system has undergone considerable development in the last two decades, with the objective to improve both its accountability and efficiency [5]. Although its physician to population ratio is within the European average [6], the perceived lack of medical doctors across Portugal has been recurrently discussed in the national press [7].

The present study analyses the characteristics of the physician population in Portugal and their home residence, with the objective to identify specific factors influencing their geographical distribution, looking specifically at the possible motivations behind foreign physicians’ choices. First, a brief review of the literature is offered on the issue of physicians’ geographical location worldwide and in Portugal, followed by a description of the methods for data collection and analysis. The results section is organised in two parts, presenting first our findings on physician characteristics and distribution across the country, and then on those municipality and demographic characteristics associated with residence location. The paper closes with a discussion on strengths and weaknesses of the present work, as well as on its policy implications and areas of further research for governments aiming at addressing physician geographical distribution unbalances worldwide.

### Literature review

One strand of the literature on physicians’ geographical location discusses whether Standard Location theory – an economic theory explaining producers’ distribution through demand factors – applies to physicians too [8]. Newhouse *et al.* (1982) argue that, although they may be able to influence demand for their own services, physicians still act as economic agents in their location behaviour, and tend to select larger cities because of the greater demand for health care services [9]. It has been elsewhere suggested that, in selecting location, physicians not only seek to maximize profit, but also take into account utility-related factors, such as leisure opportunities and proximity to urban centres [10].

From the public health perspective, a recent study across Brazilian States found a positive correlation between doctors per population and places offered in residency programmes [11]. Similar conclusions were reached by García-Perez *et al.* (2009), who found that provincial training capacity played a fundamental role in physicians’ distribution in Spain [12]. In Portugal medical doctors’ distribution has been linked to the location of National Healthcare System Institutions in which the vast majority of them are employed, and to local purchasing power [13].

Looking specifically at foreign doctors, a 1998 study in the US showed that International Medical Graduates (IMGs) constituted a greater proportion than US primary care physicians in rural areas with physicians’ shortages [14]. By the same token, Mick *et al.* (2000) found that IMGs in the US were located disproportionally in disadvantaged areas [15]. The authors’ interpretation of such finding was that, rather than exacerbating the existing surplus of national medical graduates, foreign physicians predominantly locate in areas left uncovered, thus representing a ‘safety net’ for health care service provision in the US. Other scholars [16] appear to disagree, taking the view that IMGs are not more likely than national graduates to practice in rural areas, although they tend to concentrate in specialties less suitable to urban practices. Ferrinho *et al.* (2009) note that in 2008 foreign doctors in Portugal belonged predominantly to two clusters; the main one including doctors born and trained in low-income countries with physician density lower than the Portuguese (mostly from former Portuguese colonies), and a much smaller cluster from wealthier and physician-rich countries [17].

In Portugal there were eleven medical training institutions in 2011: seven fully fledged Medicine Faculties in Lisbon, Coimbra, Oporto, Braga and Covilhã; two other Universities offering only basic courses (Açores and Madeira) and two new medicine schools (Algarve and Aveiro), accepting students with a previous university degree. After university, all graduates undertake a residency to acquire specialisation. Physicians are licensed by the Portuguese Medical Council (PMC), and there are three main medical career streams: (a) General practice/Family medicine; (b) Public Health, and; (c) Hospital-based practice (the latter including 45 specialties). The Government is jointly responsible with the PMC for the accreditation and certification of specialist training for medical graduates. Autonomous practice of medicine is only allowed (by the PMC) after two years of residency [18].

Martins *et al.* (2007) note that demand for physician services in Portugal seems to persistently exceed supply, as many professionals work overtime and for different employers at the same time [19]. However, other authors have highlighted how density of physicians in Portugal sits above the European average [20,21]. It is increasingly accepted that, rather that a problem of absolute scarcity, Portugal is currently confronted with an issue of imbalanced distribution of physicians, as the vast majority of specialised and unspecialised doctors locate in the greater Lisbon and Oporto areas, that also offer greater opportunity for better paid private practice, leaving the rest of the country grossly underserved [7]. In the light of such scenario, only lately has the Portuguese Government started encouraging physician immigration from abroad, with the expectation to fill the geographical and specialty gaps left by national doctors [22]. As recently as 2009, a much discussed decision was taken to recruit primary care physicians from Uruguay, Chile and Cuba to respond to temporary shortages in touristic rural areas in the south of the country [23].

## Methods

A cross-sectional observational study of physicians registered in Portugal was conducted with the objective to: (a) describe and analyse the physician population, and (b) explore the association of their geographical residence with relevant municipality and personal characteristics. Although this kind of studies is unable to prove the cause-effect relationship between variables, they are widely deemed valid to give a snapshot of the current situation, and explore the association between variables [24]. Existing guidelines on observational studies were followed to report the study findings [25].

The study focuses on the supply side of medical services, and its objective is to understand what attracts physicians to specific municipalities, and specifically foreign ones, with the ultimate aim of creating an evidence base for policy-makers aiming at attracting physicians in underserved areas. The underlying assumption of the research is to check the validity for the Portuguese physicians case of economic theories on physician location and of foreign physicians as a ‘safety net’ for distribution gaps.

A database was obtained from the PMC, providing information on the totality of the physicians registered in Portugal in 2008. The database included only aggregated data accordingly to variables such as physicians’ address, nationality, specialty, age, and country of training. Municipality of residence was considered a proxy for physician practice location. Besides having been used extensively in the literature on International Medical Graduates in the US [10,26], in the Portuguese case, data from the National Medical Council are considered a reliable and complete source, and often feed Government statistics on physician activities (e.g. data from the Ministry of Health or from the Ministry of Labour).

Data on municipality characteristics were obtained from the Portuguese National Statistics Institute (INE), providing information on the 2008 population, number of nurses, number of hospital beds, proportion of foreigners applying for residency in the municipality, and on 2007 purchasing power per capita. Number of beds and nurses were included as proxies of health infrastructures in the municipalities. The Municipality Development Index (MDI) was used to evaluate quality of life in specific municipalities in 2008. The MDI is a weighted welfare index developed by Municípia SA©, covering areas such as municipality’s population growth, public services, environment, economic activities and infrastructures [27].

In order to analyse physician geographical distribution, municipalities were grouped according to the INE Nomenclature of Territorial Units for Statistics of greater metropolitan areas, including 18 municipalities for the Lisbon area, and 16 for the Oporto region (see Additional file 1: Table  S1). To investigate the impact of teaching hospitals (TH) on physicians’ residence, municipalities were also divided between those in the proximity of a TH (i.e. with a TH in its territory or in a bordering municipality), and those further away. In order to analyse the impact of Medical Schools’ location on physicians’ geographical distribution, municipalities were divided into those located in proximity (around) the 10 Medical School operating in Portugal at the time of the study (42 in total) and those further away (267). Specific nationality groups were created to allow the analysis of major migrant ^a^ and linguistic groups ^b^.

Basic correlation analysis was performed to check for possible problems of multicollinearity. As the correlations were not very close to 1 or −1 (maximum correlation value was 0.647) and no increase in coefficient estimates’ standard errors was detected, it was decided to maintain in the model all the variables available from our database.

The statistical analysis was performed using SPSS 18, while ArcGIS 9.3 was employed for geographical mapping. In an initial descriptive analysis, absolute and relative frequencies, and measures of central tendency and dispersion were calculated. Chi-square tests were performed to analyse the independence of variables in the study and, when the hypothesis of independence was rejected, the corresponding residual analysis was performed. In order to compare quantitative variables among the different populations in this study, Mann–Whitney-Wilcoxon tests were applied when the assumptions for the *t*-Test failed, namely homogeneity of variances (Levene test) and normality (Kolmogorov-Smirnov and Shapiro-Wilk tests).

Taking into consideration the limitation on how the original dataset was presented, we were able to construct two databases at the municipality and the aggregate individual levels in order to answer the two main objectives in this study, namely to describe and analyse the physician population, and to explore the association of their geographical residence with relevant municipality and personal characteristics. The two separate analyses are described as follows.

For the first objective, we perform a negative binomial regression where the response variable, Y, is the total number of physicians (Portuguese, foreigners or both) in each municipality and the explanatory variables, X_i_’s, are Pop, Beds_PC, PP_PC, Nurses_PC, Foreign_PC, MDI, Metro and TH defined in Table 1.

**Table 1:** Variables considered in the study’s regression models

**Variables from PMC database (aggregate individual level)**		**Variables from INE database (aggregate municipality level)**	
**Acronym**	**Description**	**Acronym**	**Description**
Phys_tot	Absolute number of physicians (Portuguese and foreigner)	Pop	Absolute number of population
Phys_PT	Absolute number of Portuguese physicians	Beds_PC	Number of beds per 1,000 inhabitants
Phys_F	Absolute number of non-Portuguese physicians	Nurses_PC	Number of nurses per 1,000 inhabitants
Lang_1	Language (Portuguese, Spanish, English, Other)	Foreign_PC	Number of foreigners applying for residence in the municipality per 1,000 inhabitants
Metro	Binary variable for Lisbon and Oporto metropolitan areas (1) Vs non-metropolitan areas (0)	PP_PC	Purchasing Power per capita
TH	Binary variable for municipality with or neighbouring a teaching hospital (1) Vs municipalities far away from a teaching hospital (0)	MDI	Municipality Development Index

The model can be written as

Y=E(Y)+ε,

with *ε* the error term, and de variable *Y* following the distribution of

$$P(Y=y|X_{1},\ldots ,X_{8},\theta )=\frac{\Gamma (y+\theta )}{\Gamma (\theta )y!}\frac{\mu^{y}\theta^{\theta }}{{\left(\mu +\theta \right)}^{y+\theta }}y=0,1,2,\ldots \text{,}$$

with E(Y)=μ,*θ* the shape parameter, Γ(.) the gamma function, $Var(Y)=\mu +\frac{\mu^{2}}{\theta }\text{,}$ and the link function

$$g(\mu )=\log_{e}(\mu )=\alpha +\beta_{1}X_{1}+\ldots +\beta_{8}X_{8}\text{,}$$

with βi's as unknown parameters.

For the second objective, the focus was on whether the variables Sex, Age_Group, Nat_Group, and Lang_1, defined in Table 1, were significant in the location of physicians. Thus, a binary logistic regression model was performed with the variables as explanatory variables and with the reference class, Y=1, whether a doctor resides outside of a metropolitan area (Y is the variable MT in Table 1). Due to the aggregation level of the database made available by the PMC, this analysis was performed in SPSS by weighting each combination of the set of variables (MT, Sex, Age_Group, Nat_Group, and Lang_1) by the number of doctors in each class. The model can then be expressed by:

$$\text{logit}(Y)=\ln\left(\frac{\pi }{1-\pi }\right)=\alpha +\beta_{1}X_{1}+\ldots +\beta_{8}X_{8}\text{,}$$

with *π* the probability of residing outside of a metropolitan area, and unknown parameters.

The selection of variables in the model was based on the forward stepwise likelihood ratio method. In the following section we present the results of these approaches to the database made available by the PMC.

## Results

### The physician population in Portugal

According to the Portuguese Medical Council (PMC) database, there were 39,473 physicians registered in Portugal in 2008, 51.1% of which were male. Doctors between 41 and 55 years constituted the modal age class (40.2% of total), with 28.6% of the total physicians population being younger than 40 years of age and 11.9% over 65.

The vast majority of physicians were Portuguese (90.5%), with the Spanish representing the second largest nationality group (4.9%), followed by the Brazilians, other Europeans and doctors from African Portuguese-speaking (PALOP) countries. Portuguese doctors were slightly more represented among the female population, with males prevalent among the Spanish group (Table 2).

**Table 2:** Physicians registered in Portugal in 2008 by sex and nationality group

	**Female**		**Male**		**Total**	
Portuguese	17677	(91.7%)	18060	(89.4%)	35737	(90.5%)
Spanish	794	(4.1%)	1155	(5.7%)	1949	(4.9%)
Brazilian	233	(1.2%)	329	(1.6%)	562	(1.4%)
Other European	287	(1.5%)	354	(1.8%)	641	(1.6%)
African PALOP	221	(1.1%)	208	(1.0%)	429	(1.1%)
Other	64	(0.3%)	91	(0.5%)	155	(0.4%)
Total	19276	(100%)	20197	(100%)	39473	(100%)

Source: Portuguese Medical Council (2009).

**Figure 1:**
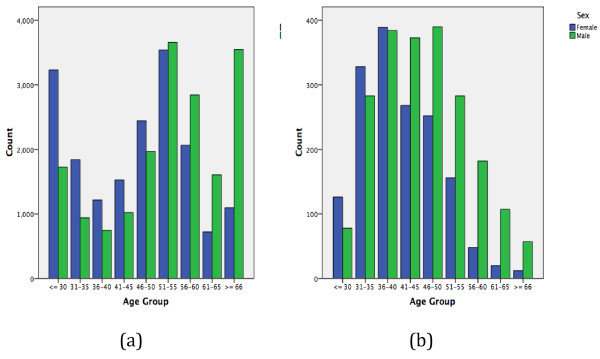
Portuguese (a) and foreign physicians (b) sex and age distribution.

Sex distribution and age composition varied considerably between national and foreign doctors, with the latter being in proportion younger and in greater proportion male than the former (50.5% Vs 57.2%) (*p* < 0.001). Feminization of the physicians workforce was most noticeable in younger age groups in both Portuguese and foreign population; before 51 years of age among the former, and before 41 in the latter (Figure 1).

General practice (also know as ‘family practice’ in Portugal) was the most common specialty among Portuguese and foreign physicians, followed by Internal Medicine and Paediatrics. An unusually large share of physicians appeared to have registered with the PMC as non-specialised (37.7%), with a disproportionate share of foreigners (72.78%) among them.

**Figure 2:**
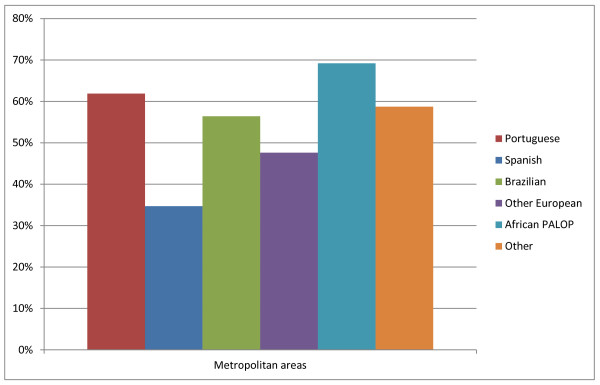
Proportion of physicians residing in metropolitan areas.

In terms of geographical distribution, both Portuguese and foreign physicians located overwhelmingly in the Lisbon and Oporto metropolitan areas, with only 39.7% of all doctors residing in the rest of the country (Table 2). In comparison with their foreign peers, Portuguese doctors showed a greater preference for being based in metropolitan areas (38.8% in Lisbon and 23.1% in Oporto Vs 26.5% and 18.7% respectively for foreign doctors), although such tendency was even more marked among physicians from African PALOP countries (52.2% in Lisbon and 17.0% in Oporto) (Figure 2).

Spanish physicians appeared to counter the generalised preference for metropolitan areas, locating predominantly (65.3%) in areas away from the two major cities, possibly closer to the border with Spain.

When looking at the physician to population ratios, metropolitan areas benefitted from having a mean of 5.30 physicians per 1,000 inhabitants, while the ratio was 2.47 outside Lisbon and Oporto areas (*p* < 0.001) (Additional file 1: Table  S2). In fact, when looking at the differences among the ratios of physicians per 1,000 inhabitants for municipalities within and outside metropolitan areas, these were found to be statistically significant (*p* < 0.001) when applied the Mann–Whitney-Wilcoxon test (*t*-Test was not applied because both assumptions of normality and homogeneity of variances failed).

**Figure 3:**
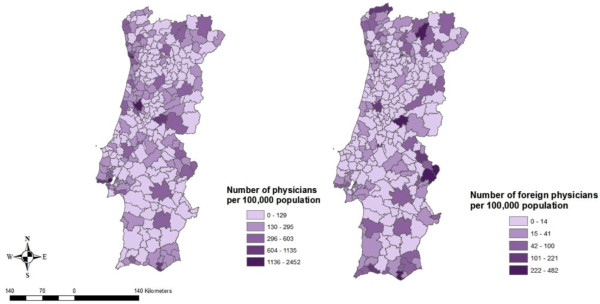
Overall and foreign physician to population ratios per municipality.

Municipalities in the proximity of a teaching hospital enjoyed 7.32 physicians per 1,000 inhabitants, as opposed to 1.97 physicians in those municipalities further away (Additional file 1: Table  S2). Again, when looking at the differences among the ratios of physicians per 1,000 inhabitants for municipalities close and away of a teaching hospital, these were found to be statistically significant (*p* < 0.001) when applied the Mann–Whitney-Wilcoxon test (Figure 3).

Geographical mapping of physician to population ratios showed a physician distribution pattern somewhat different from that of the general population. Overall physician to population ratios resulted higher for Lisbon and Oporto and for those municipalities close to a teaching hospital, while foreign physicians appear to concentrate around teaching hospitals and on few municipalities close to the Spanish border.

### The impact of municipality and physicians characteristics on physicians distribution

Negative binomial regression analysis was employed to determine what municipality characteristics had an influence on the number of physicians per municipality, in other words, what factors were statistically significant in doctors’ residence decisions. The results from the negative binomial regression modeling are shown in Table 3 below. In all the three models analysed, the omnibus test’s p-value was < 0.001 indicating that the proposed models outperforms the null model (with no predictors).

**Table 3:** Negative binomial regression of municipality characteristics on all physicians distribution

(a)				
	**Odds Ratio**	**p-value**	**95% Confidence Interval**	
Sex(Male)	1,150	0,046	1,002	1,319
Age_Group_5(>50)	1,785	< 0,001	1,507	2,113
Nat_groups_Spanish		< 0,001		
Nat_groups_Brazilian	0,307	0,003	0,139	0,677
Nat_groups_Other European	0,465	0,053	0,214	1,011
Nat_groups_PALOP	0,231	0,199	0,090	0,443
Nat_groups_Other	0,324	< 0,001	0,217	0,483
Lang_Portuguese_PALOP		0,804		
Lang_Spanish	0,863	0,706	0,402	1,855
Lang_English	1,124	0,726	0,586	2,155
Constant	1,588	< 0,001		
**n (Nagelkerke R-Square)**	**3736 (0,099)**			
(b)				
	**Odds Ratio**	**p-value**	**95% Confidence Interval**	
Sex(Male)	1,261	< 0,001	1,207	1,318
Age_Group_5(>50)	1,002	0,935	0,959	1,047
Constant	0,546	< 0,001		
**n (Nagelkerke R-Square)**	**35737 (0,004)**			
(c)				
	**Odds Ratio**	**p-value**	**95% Confidence Interval**	
Sex(Male)	1,249	< 0,001	1,198	1,303
Age_Group_5(>50)	1,041	0,066	0,997	1,086
Nat_groups_Portuguese		< 0,001		
Nat_groups_Spanish	3,450	0,002	1,605	7,416
Nat_groups_Brazilian	1,243	0,012	1,050	1,471
Nat_groups_Other European	1,772	< 0,001	1,511	2,079
Nat_groups_PALOP	0.735	0,004	0,598	0.904
Nat_groups_Other	1,189	0,604	0,618	2,289
Lang_Portuguese		0,564		
Lang_Spanish	0,886	0,755	0,415	1,893
Lang_English	1,309	0,413	0,687	2,492
Constant	0,537	< 0,001		
**n (Nagelkerke R-Square)**	**39473 (0,025)**			

The most general model set out to regress the distribution of all the physicians in the country’s municipalities on population, beds per 1000 inhabitants, purchasing power per capita, number of foreigners applying for residence per 1000 inhabitants, nurses per 1000 inhabitants, Municipality Development Index, and two binary variables - Metropolitan Areas and Teaching Hospital Areas ^c^.

Model 1: Total number of physicians on the variables Pop, Beds_PC, PP_PC, Nurses_PC, Foreign_PC, MDI, Metro, TH

In this model Population, Purchasing power per capita and Nurses per 1000 inhabitants were all significant variables (*p* < 0.05) (Table 3). Municipality Development Index (MDI) had a *p* = 0.056, i.e. very close to the 5% significance level considered in this study. “Number of foreigners applying for residence” and “Beds per 1000 inhabitants” were not statistically significant variables (*p*-value = 0.762 and 0.394, respectively).

It was interesting to notice that neither the Metropolitan nor the Teaching Hospital variables were statistically significant, possibly because their effects was picked up by the Population variable. Performing the same analysis without the variable Population, both the binary variables became statistically significant (*p* < 0.001) where, as expected, being in a metropolitan area and in or close by a teaching hospital contributed positively to the number of doctors in a municipality, although this latter model was found to have a lower explicatory power.

The second model set out to regress the same variables on the distribution of foreign physicians only across municipalities.

Model 2: Total number of foreign physicians on the variables Pop, Beds_PC, PP_PC, Nurses_PC, Foreign_PC, MDI, Metro, TH

In comparison with the previous models the differences were that: (a) Foreigners applying for residence per 1000 inhabitants was now a statistically significant variable in the model contributing positively to the number of doctors, and; (b) MDI was now clearly a non-significant variable (see Table 3).

When running the model on Portuguese physicians (our third model), as expected, MDI turned back positive and statistically significant (*p* = 0.01); PP_PC appeared to show a stronger effect on the national physicians’ distribution, while the number of new foreign applications for residence per 1000 inhabitants was no longer statistically significant (*p* = 0.296) (see Table 3).

Model 3: Total number of portuguese physicians on the variables Pop, Beds_PC, PP_PC, Nurses_PC, Foreign_PC, MDI, Metro, TH

As for the association of physician characteristics with their residence decisions, as residents in metropolitan areas appeared to enjoy higher physician to population ratios (see descriptive statistics section), in our methodology logistic regression analysis was performed to explore the effect of physician characteristics on their decision to locate outside Lisbon and Oporto metropolitan areas.

In our fourth regression model we set out to investigate the influence on the odds of physicians residing outside metropolitan areas of covariates such as sex, age, nationality and language. The assumption of physicians’ scarcity in rural areas behind our logistic model is justified by previous work on the subject [13], as well as by the Government’s efforts to recruit foreign physicians to work in rural areas [22].

Unfortunately, the binary logistic regression analysis of the variable Metro for all and the Portuguese physicians on the variables Sex, Age_Group, Nat indicating that these models were a very poor fit to the data. The only logistic regression with a Hosmer and Lemeshow _Group, and Lang_1 result in a Hosmer and Lemeshow test with a *p*-value < 0.001 test with a *p*-value > 0.05, indicating that this model was a good fit to the data, is shown next, and it is relative to foreign physicians only, with age group divided by > 50 and < = 50 years old:

Model 4: Binary logistic regression of the variable Metro for all physicians on the variables Sex, Age_Group, Nat_Group, and Lang_1 (Table 4)

**Table 4:** Logistic regression for probability to reside outside metropolitan areas for foreign physicians

	**Odds ratio**	**p-value**	**95% Confidence interval**	
Sex(Male)	1,150	0,046	1,002	1,319
Age_Group_5(>50)	1,790	< 0,001	1,513	2,119
Nat_groups_Spanish		< 0,001		
Nat_groups_Brazilian	0,356	< 0,001	0,292	0,433
Nat_groups_Other European	0,541	< 0,001	0,450	0,651
Nat_groups_PALOP	0,231	< 0,001	0,184	0,290
Nat_groups_Other	0,344	< 0,001	0,245	0,482
Constant	1,588	< 0,001		

Being male and being 50 or more years of age increased the likelihood of a foreign physician residing outside metropolitan areas. The Language variable (i.e., the ability to speak Portuguese) was not significant and therefore dropped from the model.

As for nationalities, in comparison to the reference category “Spanish”, belonging to any other nationality decreased the likelihood of finding a physician outside Lisbon and Oporto areas. It is worth noticing that African PALOP physicians resulted the nationality group less likely to be found outside the two metropolitan areas, while the “Other European” group behaved in the same direction as the reference category of “Spanish” doctors.

## Discussion

The present study provides a thorough analysis of physician population in Portugal, and showed that the physicians registered in the country are almost equally male and female, mostly Portuguese and between 41 and 55 years of age. Spanish, Brazilian, PALOP and other European nationalities are also a significant presence among foreign physicians. A considerable unbalance was detected in the physician-to-population ratio between metropolitan areas and the rest of the country, favouring particularly those municipalities in the proximity of a teaching hospital. Among the variables considered, population, nurses and purchasing power per capita were the most important municipality characteristics associated with physician distribution in Portugal. For national doctors, the Municipality Development Index was significant too, and so was for international doctors the proportion of foreigners applying for residence. Foreign physicians resulted, in proportion, more likely than the Portuguese to be found outside metropolitan areas. However, such effect appeared to be mostly due to the Spanish cohort, as other nationalities – especially the African PALOP - displayed location preferences similar to those of the Portuguese. Among foreigners, males and over-50s were more likely to reside outside the Lisbon and Oporto areas. Being able to speak Portuguese or Spanish as mother tongue did not seem to carry further influence on location decisions beyond nationality.

A number of limitations need to be considered when interpreting the results of our study. First of all, our ‘physician residence’ endpoint does not necessarily correspond to physicians' geographical area of practice, although it may arguably provide a good indication of it. Due to privacy concerns, the PMC database employed for our analysis did not disclose information on individual physicians, and this limited considerably the statistical analysis that could be performed. As many physicians appeared to be registered under more than one key characteristics such as specialty or years practice, this made it impossible to apply our statistical methods to explore potential explanatory variables of physician location.

A further point is that the cross-sectional nature of our data limited inference on the cause-result relationships between physicians’ residence and municipality characteristics. However, as our dataset used the most reliable and comprehensive source of information in Portugal, and as similar sort of data have been used in the past in similar studies, the authors still consider the results obtained a fair representation of physician population in Portugal. There is recognition of a need for more complete municipality data to be included in our analysis as proxies for need, such as mortality, morbidity and epidemiological data. As the impact of these may well not be fully captured by the population variable. To this respect, a request to relevant sources has been already made, and a plan exists to make use of a more complete set of variables for future work. Finally, the logistic regression model on physician characteristics to investigate the odds of residing outside metropolitan areas could only be employed for the foreign physicians population, as it displayed a poor fit for the larger Portuguese physicians cohort.

Despite its limitations, a few considerations can be safely drawn from our study. The study confirms the conventional wisdom from the literature that physicians distributions is related to the distribution of infrastructures - here represented by nurses and beds - and by population affluence –represented by MDI and PPP per capita -, possibly exacerbating the imbalance between poor, underserved areas and rich and well-provided ones. However, the study findings also suggest that physicians reside disproportionally in metropolitan areas, and that factors influencing their geographical residence may be in some respect different for national and international doctors. This appears to be consistent with the literature on international medical graduates [11,13,16]. For national doctors, the relevance of those municipality characteristics linked to supply-side variables– such as the MDI and PP_PC – as well as their concentration in the Lisbon and Oporto areas, may be signalling a higher discretionary power enjoyed in selecting residence location [28]. For foreign physicians, the importance of more demand-related variables - such as hospital beds and presence of foreign population – as well as their stronger presence outside metropolitan areas, may be interpreted as a more pronounced dependency of foreign physicians on public sector employment opportunities, as well as a lesser access to premium locations. If this were the case, incentives to locate in under-served rural areas may be effective policy options to redistribute Portuguese physicians, either through better public sector salaries or linked to the possibility of developing their own private practice. To foreign doctors, incentives linked to possibility to practice among, or in the proximity of, their ethnic community of origin may represent sensible solutions to attract this type of professionals in the long run.

A closer investigation of foreign physician distribution also showed that the claim that these can be used indiscriminately to fill geographical gaps is somewhat inaccurate, as rural residence patterns appeared to vary considerably among nationality groups, with PALOP physicians concentrating in metropolitan areas even more than their national peers. Such finding resonates with the existing literature on foreign physicians in the United States [16], and its implication would be that, relying on international recruitment might not be the most adequate policy to address physician shortages in rural areas. To this respect, policy-makers may consider alternative policy options such as:

• Delegating basic medical acts to nurses and other professionals for rural areas [29,30];

• Creating incentives to enrol professionals in rural-areas-focused programs such as non-hospital-based specialties general practice (family medicine) and in public health/community medicine [31], or;

• Expanding the teaching hospital network in rural areas, as the experience shows that locally recruited and trained professionals are less likely to leave to metropolitan areas [32].

## Conclusions

The present study investigated physicians’ population and residence location across Portugal, with the objective of gaining a better understanding of the factors influencing their distribution across the country. Through descriptive and regression analysis, the study identified some of the municipality and physician characteristics displaying association with medical doctors’ distribution and their decision to locate outside metropolitan areas.

The study showed the value of collecting and analysing data on physicians and municipality characteristics, in order to design effective policies to address distribution imbalances across within a country. The study also highlighted the need for better and more detailed data on physician distribution to inform policies addressing health personnel geographical disparities countrywide, possibly through longitudinal studies.

Evaluating the impact of such policies would be an area for further research, as limited evidence seems to exist on the subject, especially with reference to the Portuguese case [33]. Qualitative investigation of physicians residence choices would also help shed light on which of these policies may be more effective than others, and under what conditions [34]. Discrete choice experiments may too provide an insight into those strategies to attract and retain health professionals not yet tested in Portugal or in a similar policy context [35].

## Endnotes

^a^ Portuguese, Spanish, Brazilian, Other European, African Portuguese-Speaking Countries – PALOP.

^b^ Portuguese-speaking countries, Spanish-speaking ones, European, and Others.

^c^ University of Açores, University of Beira Interior, University of Coimbra, University of Lisboa, New University of Lisbon, University of Madeira, University of Minho, University of Oporto (2 medical schools), and University of Algarve. The newly inaugurated Aveiro Medical School was not operational at the time of the study and thus not considered to construct the teaching hospital variable.

## Competing interests

The authors declare that they have no competing interests.

## Authors’ contributions

GR participated in the design of the study, in the data collection and analysis, and carried out the drafting of the manuscript. PF participated in the design of the study and in the manuscript drafting. BdS carried out the statistical analysis of the data and participated in the drafting of the manuscript. CC participated in data collection and in drafting the manuscript. All authors read and approved the final manuscript.

## Supplementary Material

Additional file 1 Statistical annex. Click here for file
