# Supplementary material for: What influences national and foreign physicians’ geographic distribution? An analysis of medical doctors’ residence location in Portugal
Source: Hum Resour Health. 2012 Jul 2;10:12. doi: 10.1186/1478-4491-10-12 (PMC3549747; doi:10.1186/1478-4491-10-12)
Supplement: Additional file 1 — Statistical annex. [file 1478-4491-10-12-S1.doc]

# Statistical annex

Table 1: Municipalities considered in the main metropolitan areas

| Lisbon metropolitan area | Amadora, Cascais, Lourés, Mafra, Odivelas, Oeiras, Sintra, Vila Franca de Xira, Alcochete, Almada, Barreiro, Moita, Montijo, Palmela, Seixal, Sesimbra, Setubal. |
| --- | --- |
| Oporto metropolitan area | [Arouca](http://en.wikipedia.org/wiki/Arouca,_Portugal), [Espinho](http://en.wikipedia.org/wiki/Espinho), [Gondomar](http://en.wikipedia.org/wiki/Gondomar_(Portugal)), [Maia](http://en.wikipedia.org/wiki/Maia,_Portugal), [Matosinhos](http://en.wikipedia.org/wiki/Matosinhos), [Oliveira de Azeméis](http://en.wikipedia.org/wiki/Oliveira_de_Azeméis), [Porto](http://en.wikipedia.org/wiki/Porto), [Póvoa de Varzim](http://en.wikipedia.org/wiki/Póvoa_de_Varzim), [Santa Maria da Feira](http://en.wikipedia.org/wiki/Santa_Maria_da_Feira), [Santo Tirso](http://en.wikipedia.org/wiki/Santo_Tirso), [São João da Madeira](http://en.wikipedia.org/wiki/São_João_da_Madeira), [Trofa](http://en.wikipedia.org/wiki/Trofa), [Vale de Cambra](http://en.wikipedia.org/wiki/Vale_de_Cambra), [Valongo](http://en.wikipedia.org/wiki/Valongo), [Vila Nova de Gaia](http://en.wikipedia.org/wiki/Vila_Nova_de_Gaia) and [Vila do Conde](http://en.wikipedia.org/wiki/Vila_do_Conde). |

Table 2: Population and physicians per type of area

| Geographical area | Number of municipalities | Population | Mean population | Number of physicians* | Physician per 1000 inhabitants |
| --- | --- | --- | --- | --- | --- |
| Metropolitan areas | 34 | 4 495 074 | 132 208 | 23 814 | 5.30 |
| Rest of the country | 274 | 6 127 338 | 22 363 | 15 118 | 2.47 |
| Municipalities neighbouring TH | 41 | 3 364 320 | 82 057 | 24 612 | 7.32 |
| Municipalities away from TH | 267 | 7 258 092 | 27 184 | 14 320 | 1.97 |
| Total Portugal | 308 | 10 622 412 | 34 488 | 38 932 | 3.67 |

*Excluding physicians living abroad or recording no residence information

Table 3: Hosmer and Lemeshow test for logistic regression model

| Chi-square | df | Sig. |
| --- | --- | --- |
| ,000 | 1 | 1,000 |
| 11,213 | 5 | ,047 |
| 49,035 | 8 | < 0,001 |

Table 4: Hosmer and Lemeshow test and contingency tables for Logistic Regression model

| Chi-square | df | Sig. |
| --- | --- | --- |
| 1,604 | 5 | ,901 |

Table 4: Descriptive statistics per area variables

| **Variable** | **Type of geographical areas** | | | | | |
| --- | --- | --- | --- | --- | --- | --- |
|  | **Metropolitan** | | | **Not metropolitan** | | |
|  | **Sum** | **Mean** | **SD** | **Sum** | **Mean** | **SD** |
| Population | 4,495,074 |  |  | 6,127,338 |  |  |
| Number of physicians |  |  |  |  |  |  |
| Portuguese doctors | 22,128 |  |  | 13,367 |  |  |
| Foreign doctors | 1,686 |  |  | 1,751 |  |  |
| Unknown location | 541 | | | | | |
| Beds per municipality |  | 404 | 1,299 |  | 41 | 227 |
| Population Purchasing power |  | 110.83 | 34.91 |  | 70.45 | 18.92 |
| Foreign residence applications per 1000 hab. |  | 578 | 370 |  | 497 | 771 |
| Municipality development index |  | 134.43 | 103.16 |  | 95.73 | 38.64 |
|  | **Sum** | | **Row N %** | **Sum** | | **Row N %** |
| Sex |  | |  |  | |  |
| Female | 12,215 | | 46.60% | 7,061 | | 53.40% |
| Male | 11,599 | | 42.80% | 8,598 | | 57.20% |
| Age groups |  | |  |  | |  |
| <= 35 | 5,093 | | 47.30% | 3,458 | | 52.70% |
| 36-45 | 3,652 | | 51.50% | 2,271 | | 48.50% |
| 46-55 | 7,332 | | 40.70% | 5,364 | | 59.30% |
| 56-65 | 4,480 | | 39.90% | 3,111 | | 60.10% |
| > 65 | 3,257 | | 42.50% | 1,455 | | 57.50% |
| Nationality |  | |  |  | |  |
| Portuguese | 21,706 | | 42.50% | 13,367 | | 57.50% |
| Spanish | 757 | | 37.10% | 1,365 | | 62.90% |
| Brazilian | 440 | | 62.90% | 258 | | 37.10% |
| Other European | 473 |  | 53.30% | 421 |  | 46.70% |
| African PALOPs | 208 |  | 78.20% | 54 |  | 21.80% |
| Other | 230 |  | 53.50% | 194 |  | 46.50% |
